# THE ETHICS OF SHAM SURGERY IN PARKINSON'S DISEASE: BACK TO THE FUTURE?

**DOI:** 10.1111/j.1467-8519.2011.01931.x

**Published:** 2011-12-13

**Authors:** Teresa Swift, Richard Huxtable

**Keywords:** sham surgery, research ethics, Parkinson's Disease, placebo, neurosurgery

## Abstract

Despite intense academic debate in the recent past over the use of ‘sham surgery’ control groups in research, there has been a recent resurgence in their use in the field of neurodegenerative disease. Yet the primacy of ethical arguments in favour of sham surgery controls is not yet established. Preliminary empirical research shows an asymmetry between the views of neurosurgical researchers and patients on the subject, while different ethical guidelines and regulations support conflicting interpretations. Research ethics committees faced with a proposal involving sham surgery should be aware of its ethical complexities. An overview of recent and current placebo-controlled surgical trials in the field of Parkinson's Disease is provided here, followed by an analysis of the key ethical issues which such trials raise.

## INTRODUCTION

In 1999 neurosurgical researchers^1^ in the United States announced their intention to conduct a federally funded Phase 2 randomized controlled trial to test the experimental intervention of fetal dopaminergic cell transplantation (FCT) in patients with Parkinson's Disease (PD) using a stereotactic surgical technique.^2^ What provoked controversy was their intention to use a ‘sham surgery’ control group. Patients randomized to the control group would undergo all the procedures associated with the real surgery except for the surgical manoeuvre itself. This would involve two operations, each of which would include: the attachment of a stereotactic frame to the skull; MRI and PET scans; general anaesthesia; skin incisions; the drilling of a partial burr hole into the skull (not penetrating the brain), follow-up antibiotics and six months of immunosuppressive therapy.

Heated ethical debate ensued. Nonetheless, trials utilizing a sham surgery group continue to be conducted in the United States, supported by the ethical guidelines of particular professional bodies,^3^ while a sham-controlled trial in the field of osteoarthritis was recently conducted in the United Kingdom.^4^ Various general guidelines^5^ andEuropean legislation^6^ on the protection of human subjects are stringent in relation to the primacy of individual research participants' best interests but they do not provide an absolute prohibition against sham surgery and are thus subject to interpretation. In light of these factors it would seem prudent for research ethics committees to remain abreast of the rare but regular conduct of sham surgery and for them to be well informed about the ethical complexities inherent in the design. With this in mind, we offer an overview of recent and current sham-controlled trials in the field of PD and an analysis of the key ethical issues and conflicts which such trials raise. We write from the UK perspective, but much of what we say will be widely applicable.

The discussion will be structured using Foster's ‘three-approaches’ framework for the ethical review of research, according to which ethics committees are invited to consider the goal-based, duty-based and right-based issues arising in a particular project.^7^ It is, therefore, necessary to start with an outline of the projects in question i.e. those PD trials in which sham surgery controls have been employed.

### Parkinson's Disease, Treatment and Sham Surgery

The ethical issues surrounding sham surgery will doubtless also be relevant outside the sphere of neurodegenerative disease.^8^ Nevertheless, we focus here on trials involving PD since it was trials for this condition which generated much of the ethical debate over the past decade, and because this is a field in which sham surgery continues to be used. Particular ethical issues may arise, however, as a consequence of certain features of PD such as the degenerative nature of the condition and the present lack of treatment to cure it or halt its progression.

Named after James Parkinson, following his 1817 monograph *An Essay on the Shaking Palsy*, Parkinson's disease (PD) is a degenerative movement disorder caused by neurological dysfunction. Two of the following four main features should be present before a definitive diagnosis is made: resting tremor; rigidity; paucity of movement, including slowness and freezing, and loss of postural reflexes.^9^ Standard treatment is pharmacological. Levodopa is currently the most effective drug treatment for PD; however, not every patient will respond to levodopa and those who do are likely to experience unwanted side-effects after several years, including severe dyskinesia, such as peak-dose or end-of-dose deterioration,^10^ and psychiatric disturbance.^11^ Other drugs include dopamine agonists, MAO-B inhibitors and COMT inhibitors.^12^

In addition to the neurotransplantation technique described above, other surgical approaches to PD include ablative surgery and deep brain stimulation (DBS) which have both been found to improve symptoms in some patients but with differing degrees of success. DBS has now been approved by US Food and Drug Administration (FDA), hence it is reasonable to ask why it might not be used as a standard treatment comparison in a controlled trial of FCT. It is, however, not a cure for PD but performed to manage abnormal movement symptoms. In contrast, FCT is aimed at addressing the underlying degeneration of dopamine-producing neurons which cause the symptoms of PD.

Results of a completed sham-controlled study of FCT in PD^13^ were published in 2001. This study involved 40 patients with severe PD, 20 of whom were in the placebo arm. Aggregate risks were reduced for the sham group in this trial by the use of local anaesthesia and no post-operative immunosuppression. Nonetheless, a stereotactic ring was still affixed to all participants' skulls and they received MRI and PET scans, scalp incision and four partial twist drill holes in the skull. The study found that the fetal dopamine neurons survived in the putamen of all the patients in the experimental group. However, there were no statistically significant differences between the disease ratings for the two groups, although patients under 60 did show (and report) significantly greater improvements than those in the sham group. The real surgery was thereafter offered to those in the sham group, and 14 took up the offer before the emergence of undesirable symptoms in five patients who had the real surgery prompted the scientists to advise the remainder against proceeding. These patients had initially seen improvements but they later experienced ‘runaway dyskinesias’, which remained even when their usual levodopa medication was stopped. The scientists hypothesised that this may have been attributable to continued fibre outgrowth from the transplant leading to a relative excess of dopamine production.

The results of Freeman, Olanow and colleagues' FCT study, described earlier, were published in 2003. 34 patients were recruited into this trial, 11 of whom received placebo operations.^14^ The results were disappointing; indeed, 56% of the transplant recipients developed dyskinesias, which persisted even after medication was stopped. The authors hypothesized that this was due to ‘partial, but inadequate, graft survival’,^15^ sufficient to release low levels of dopamine for a prolonged period of time, but not sufficient to produce an anti-Parkinsonian response. They concluded that fetal nigral cell transplantation could not at that time be recommended for PD.

Although there were other contemporaneous PD trials employing sham surgery methodology,^16^ the studies described above generated most debate. Yet, by 2009, even these scientists appeared pessimistic about the prospects for FCT. As Olanow et al. concluded:

The failure of dopaminergic cell-based therapies to achieve efficacy in double blind clinical trials, the development of unanticipated and occasionally disabling side effects, evidence that implanted cells themselves can develop the pathological changes of PD, and the likelihood that these treatments will not address the nondopaminergic features of the disease do not bode well for the near-term future of cell-based therapies as a clinically meaningful treatment for the majority of patients with PD.^17^

A recent study, however, offers new hope for the procedure; reporting that the systemic administration of a serotonin receptor agonist, which dampens transmitter release from serotonergic neurons, can result in a ‘marked reduction’ in dyskinesias.^18^

There are also developments in the field of gene therapy which suggest that sham surgery for patients with PD is far from being consigned to history. Phase 2 of a study conducted by Ceregene (a private biotechnology company based in San Diego) used a double-blind, sham controlled design to investigate the efficacy of a potential gene therapy entitled CERE-120.^19^ The therapy involved the delivery of neurturin^20^ to the putaminal region of the brain using stereotactic neurosurgery. 58 patients with advanced PD were enrolled, two thirds of whom received the real treatment. According to a press release in November 2008, although no harms were reported, the results were ‘disappointing’, not showing any significant differences between the two groups of participants. Despite this, another press release in April 2010 announced a new trial, which ‘builds on experience gained in that trial, by enhancing the dose regimen and optimizing the duration of patient follow up’, and will again use sham surgery in the Phase 2 stage.^21^

Sham surgery was also used in the field of PD research in the Phase 2 Spheramine trial, backed by private companies Bayer Schering Pharma and Titan Pharmaceuticals. The surgical intervention under investigation consisted of microscopic gelatin ‘beads’ onto which were bonded human retinal pigment epithelial cells which manufacture levodopa. In June 2008 the company announced that initial analyses showed that Spheramine did not meet the study's primary or key secondary endpoints, with no significant differences between the real treatment and sham.^22^

Proponents of sham surgery may use these results as evidence of the sham design's usefulness in stopping the development of techniques which have no efficacy above placebo. Concerns have been expressed, however, by a US patient advocacy group, the Parkinson's Pipeline Project, that the interim analyses which result in trials being halted as failures are being conducted too soon to allow for maturing of real treatment effects and for placebo to ‘wear off’. The group is calling for longer assessment periods in order to avoid Type 2 errors.^23^

Most recently, and more positively, biotechnology company Neurologix announced the success of another Phase 2 trial – also sham-controlled – testing an adeno-associated virus (AAV) vector gene transfer technology to deliver an inhibitory gene to alter neural circuitry in PD.^24^

## ETHICAL GUIDELINES

One could be forgiven for thinking that the recent re-emergence of sham surgery means that the ethical debate has already been won by those in favour of employing this method. Indeed, practitioners of the methodology in the United States (where these studies have all taken place) may draw on specific guidance supporting placebo controls.

The FDA requires a trial to be ‘adequate and well controlled’ and offers five acceptable control designs, namely: placebo, dose-comparison, no treatment, active treatment and historical control.^25^ More specifically, the American Association of Neurological Surgeons and the Congress of Neurological Surgeons issued a position statement in January 2000 (reaffirmed in November 2009) in favour of placebo-controlled surgical trials, stating that they ‘support the use of placebo surgery in clinical trials but under limited and carefully selected guidelines’ which include: evaluation by local committees to ensure placebo methodology is necessary; that the placebo procedure be as safe as possible, and that patients are fully informed of the nature of the study, the need for a placebo control group, and treatment alternatives.

In addition, the Council on Ethical and Judicial Affairs of the American Medical Association issued guidelines^26^ stating that as long as no other design could yield the requisite data:

When a new surgical procedure is developed with the prospect of treating a condition for which no known surgical therapy exists, or when the efficacy of an existing surgical procedure comes into question, a study design using surgical placebo controls may be justified if it is known that the disease being studied may be susceptible to a placebo effect and the risks of the surgical placebo effect and the risks of the surgical placebo control operation are relatively small.

Depending on one's interpretation of ‘relatively small’, these recommendations can be taken as supportive of sham surgery in the case of the PD trials. However, given its continuing use, we would suggest that there is still a need to consider the ethical issues associated with this method, and particularly its use in relation to patients with PD. We can best illustrate the on-going ethical difficulties by viewing them through the prism provided by Clare Foster.^27^ Writing for those involved in medical research with human subjects, Foster identifies three areas of primary ethical concern, labelling them, respectively, goal-based, duty-based and right-based. We will address each in turn.

## GOALS, METHODS AND PLACEBOS

Goal-based research ethical concerns comprise consequentialist considerations, such as the value of the research goal to society and the choice of a method which will best achieve that goal. Central to the case for sham surgical trials is the argument for the need to control for bias and the placebo effect through a scientifically rigorous double-blind, placebo-controlled trial. New surgical interventions are, generally, introduced after far less rigorous evaluation than that required for new medical interventions and previous studies using sham surgery controls^28^– dating back some decades – have revealed the inefficacy of previously accepted surgical techniques and prompted their abandonment.^29^ In addition, the placebo response may be a larger factor in this type of surgery than in more immediate life-saving procedures,^30^ although the size of the surgical placebo effect in general has been disputed.^31^ Gillett also points out that surgery is much safer than it once was; indeed, surgery may be less serious than exposing a patient to drugs that affect the entire body.

Surgery is, however, often irreversible, unlike drugs, which can be discontinued.^32^ More generally, the appropriateness of applying RCTs to surgery has been much debated,^33^ and the utility of placebo controls questioned.^34^ Dekkers and Boer believe a preferable alternative methodology exists, which they label a ‘core assessment protocol’ (CAP), in which measurement protocols are applied to a patient pre- and post-operatively.^35^ However, they concede that this approach could not completely eliminate observer and patient bias about outcome. Preference trials have been suggested as a more ethical alternative^36^ but unequal randomization may lead to loss of statistical power and trial sample size may need to be increased accordingly.^37^ Dekkers and Boer, for their part, also feel that, technically if not ethically, the sham arm of an FCT trial should involve insertion of the cannula into the brain if it is to elicit the full placebo effect.^38^ Landau make the point that brain penetration control data improves accuracy, since the resultant non-specific neural tissue injury appears, paradoxically, to lead to improvement in Parkinsonian symptoms.^39^ This would, however, introduce further risks for the participants in the sham group.

As such, there are lingering questions about the applicability of, and necessity for – and thus the goal-based justifications for – sham surgery. Nevertheless, the arguments in favour do have *prima facie* plausibility, sufficient at least to guard against ruling out such a method at this early juncture.

## DUTIES, BENEFITS AND RISKS

Matters become much less clear-cut when we turn to the more distinctively deontological considerations associated with Foster's duty-based category, in which our central concern should be for the best interests of the participant. Here, the need to balance risks to patients against the potential benefits of developing an effective therapy, or establishing that an unproven therapy is ineffective, has not gone unnoticed.^40^ The surgical procedure itself carries non-trivial risks, and inevitably postpones uptake of other options (including, if successful, the study surgery itself). Yet, say Freeman et al., the benefits are considerable:

contributing to advances in the treatment of a disease of great personal interest to the participants, receiving standard medical treatment at no cost, having the opportunity to obtain fetal-tissue transplant at no cost if the procedure proves to be safe and effective, and being spared the risks associated with transplantation if it proves to be unsafe or ineffective.^41^

According to a classification of benefits used by King, these qualify merely as collateral benefits rather than the direct benefits of the intervention under investigation.^42^ Guidelines from the US National Bioethics Advisory Commission state that only direct benefits should be included in a risk/potential benefit calculation.^43^ Nonetheless, Gillett and Freeman both believe that it may be in the individual patient's best interests to enter a trial which could halve her chance of receiving potentially beneficial treatment, since this also halves her risk of receiving the possible harms associated with that treatment.^44^ This is an appeal to the concept of equipoise. It is important to ask, however, whose equipoise counts, since that of the patient may differ considerably from that of the investigator or clinical community.^45^ Indeed, it is difficult to determine the relative weight that should be attributed to risks and benefits, given the inevitable subjectivity of – and diversity amongst – these judgements. Ethics committees are likely, therefore, to reach differing conclusions, and maybe reasonably so.^46^

Weijer argues that research should be subject to component ethical analysis in which the risks of non-therapeutic^47^ trial elements are calculated separately to those where benefit may obtain, and judged in relation to a standard of ‘minimal risk’.^48^ Minimal risk has been defined in various ways^49^ and comparisons of procedures to these definitions are subject to varying judgements. Nonetheless, for some commentators,^50^ the sham surgeries in the PD trials described above clearly fail a test of non-maleficence while Freeman et al. themselves described the risks as ‘non-trivial’. Patients may still have reason to enter a trial where the risks of diagnostic procedures outside the trial are no less than those in the placebo arm^51^ but it is debatable whether this is the case for sham surgery in PD. In sum, there is, at best, uncertainty about the extent to which sham surgery intrudes upon the best interests of the participant.

## GOALS OR DUTIES?

To return to Foster's framework, tension clearly exists between the duty to protect the participant's best interests and the goal-based concerns of research methodology. Albin provides a useful example of this tension, since he admits that the increased risks render sham surgery ‘unattractive’, but he nevertheless concludes in favour of the procedure, given the strength of the scientific arguments – albeit provided that the risks to participants are minimized.^52^ His position involves the subordination of duty-based concerns beneath the goal-based preoccupation with scientific advancement for the good of future patients.

Others prefer to favour the duty to protect patient's best interests – but quite what this obligation means is a matter of some dispute. Indeed, Franklin G Miller, writing with a succession of colleagues,^53^ resists the conflation of clinical care and clinical research trials implicit in this duty, arguing that each endeavour pursues different goals, and the latter ‘are not designed to promote the best interests of patients and often expose them to risks that are not outweighed by known potential medical benefits’. Miller generally advances the argument that the risk-benefit analysis therefore need not always be favourable to the individual patient, stating that ‘clinical trials routinely administer interventions whose risks to patients are not compensated by medical benefits but are justified by the anticipated value of the scientific knowledge that might be gained’.^54^ He concludes that such studies are generally considered ethically acceptable ‘provided risks have been minimized, are not excessive and are justified by the value of the knowledge to be gained.’^55^

The danger of this is that ‘investigators are relieved from the constraints of the personal-care principle and the absolute prohibition against doing harm’.^56^ Critics note a number of flaws in this argument. First, the goals of research do not determine the ethics of it.^57^ Second,research has a number of goals in addition to pursing scientific knowledge generation, including the aim of benefiting patients, future and current.^58^ Third, the attempt to separate research from treatment overlooks the fact that it is often doctors who are conducting the research, who are accordingly duty-bound to protect the interests of patients.^59^ Rhodes suggests sardonically that Miller's distinction can be made clearer to patients and participants, perhaps by clinicians wearing white coats, but switching to lavender when they assume their researcher role. Likewise, patients do not cease to be patients when they enter research trials, and even if they were not a patient before (which is unlikely in the case of PD), they immediately become one once they have undergone surgery.^60^

Chalmers Clark similarly warns against using patients as a means to others' ends and points to the American Medical Association's Code of Medical Ethics, which states that in a conflict, benefit to the patient outweighs other duties – which, for him, explains much of the unease surrounding sham surgery.^61^ But, argues Kim, physicians ‘not infrequently’ serve in roles in which the duty of beneficence is subordinated to another interest, such as in forensic evaluations, when the doctor's primary duty is to the state. Kim accordingly judges clinical equipoise an attractive concept for protecting patients' interests within a trial, but not an absolute requirement.^62^ Weijer resists this conclusion: he reminds us that the concept of equipoise was developed precisely in order to reassure doctors that participants would not be at risk of receiving substandard care^63^– in other words, to avoid exploitation.

## RIGHTS, AUTONOMY AND TRUST

We seem to have reached an impasse: the duty to protect patients as research participants apparently comes up against the prospect of securing accurate data regarding potential benefits for patients at large. To proponents of sham surgery, the solution will be clear: turn the matter over to the prospective participants themselves. Kantian ideals, like the obligation to treat persons as ends-in-themselves, can be observed by insisting upon rigorous standards of consent;^64^ surely once these are satisfied, there can be no barrier to proceeding? After all, the imperative does not preclude participants from being ‘used’ in research, provided that they are not *solely* used as means to an end.^65^ But what are the standards of consent that should obtain in relation to sham surgery, and are these standards attainable?

Consent conventionally comprises three essential elements: the person consenting must *competently* reach a *voluntary* decision, which is sufficiently *informed* about the procedure in question. These criteria feature in many legal systems and in philosophical accounts of respect for autonomy, such that the failure to satisfy any one criterion might render a decision non-autonomous.^66^ Foster would classify such concerns as right-based, and there is certainly a case for concluding that it should be down to the rights-bearer to determine whether or not to run any potential risks in a sham surgery trial. To conclude otherwise would be paternalistic, as Macklin acknowledges.^67^

Yet, Macklin still thinks there is cause for concern here, given the prospect of the ‘therapeutic misconception’. This is the erroneous idea that research projects are designed with the primary goal of directly benefiting participants, or that they will potentially obtain more benefit than is scientifically expected.^68^ Empirical studies have shown that some patients fail to understand information about the trial they have been invited into.^69^ They may overestimate benefits,^70^ underestimate risks^71^ or fail to understand randomization.^72^ In short, it seems that the informed consent process cannot adequately ensure comprehension, so that we cannot guarantee that participants will be sufficiently informed. We could respond by improving our efforts at ensuring comprehension, through suitably worded consent forms and information.^73^ However, although improvements in disclosure may improve this figure, ‘misunderstanding’ about research may be ‘a persistent and incorrigible feature of people's participation in research’.^74^ This may be because of the deep trust people place in research and in those who conduct it.^75^ Fletcher thinks we can trust the neurosurgeons and their teams to address unexpected or adverse events.^76^ Are participants right to trust in this way; or is trust misplaced when the risks seem so grave?

And, the sceptics continue, there is further good reason for thinking that any consent will not be as maximally autonomous as the supporters of sham surgery might hope. Here, the voluntariness^77^ of any consent that might be obtained from a participant gives some cause for concern. Aside from issues such as the power imbalance between doctor and patient^78^ and well-meaning but potentially coercive behaviour from relatives,^79^ there are questions over the autonomy of patients with PD and whether they are genuinely free to choose to participate in a research trial^80^ and hence whether they are vulnerable to accepting an exploitative research offer.^81^ Clark believes that voluntary informed consent is likely to be questionable from patients for whom the surgery represents their only hope of relief from their condition.^82^ He points out, however, that this would seem to be a general problem with recruiting participants with any incurable condition. Miller would undoubtedly argue differently, as he has cited evidence from a trial of arthroscopic surgery, which shows that allegedly desperate patients do decline to participate – and thus are probably not so desperate or vulnerable after all.^83^ Informed consent, however, is likely to be most problematic where experimental treatments are not only restricted to trial but also tested using a sham control group.^84^ Regarding the latter, many trials use comparison to a standard treatment; others use inert placebo controls. Other forms of active placebos exist^85^ but sham surgery is arguably the riskiest and most invasive type of active placebo. Regarding restriction to trial, it warrants emphasis that the arthroscopy trial cited by Miller tested a procedure that was already widely available in clinical practice.^86^ If, in contrast, a trial represents the *only* hope of obtaining benefit (in other words, there are no other efficacious treatment options), then surely this exerts some influence on a patient's decision to enrol? Nevertheless, Cohen et al.,^87^ who are members of the Parkinson's Pipeline Project, feel that clinicians ‘often mistake’ patients' sense of urgency for improved therapies to be made available for desperation, but patients view themselves as informed and realistic. Despite this, they conclude that the availability of alternative trial designs means that ‘exposing patients to the risks of sham surgery in most cases is unethical and should be avoided’.^88^

## THE ROLE OF RESEARCH ETHICS COMMITTEES

Implicit in Cohen et al's conclusion is a sense that an appeal to autonomy is insufficient. After all, the ethical review process is in place precisely to determine the acceptability of risks – and only thereafter should the matter be turned over to the potential participant. Indeed, if autonomy alone were to be determinative, then there would be little need for ethics committees.^89^ Edwards et al. have in fact argued that a research ethics committee's (REC's) role could be merely to assess goal- and right-based issues such as methodology and informed consent, in order to avoid a charge of paternalism.^90^ Such a committee would avoid the difficulties associated with ‘weighing up’ the risks and benefits to patients of participating in a trial. Garrard and Dawson argue, however, that the individual patient is not as qualified to comprehend all aspects of a trial as a panel of individuals gathered together purposefully to form a REC. This, they argue, is the source of a REC's moral authority to make decisions in patients' best interests.^91^ Foster too argues that to place the full burden of understanding a trial's risks and benefits upon a person possibly unqualified in matters of research methodology and technical, medical or surgical procedures seems inadequate protection and is possibly a step too far in defence of right-based morality at the expense of duty-based considerations.^92^

A REC concerned only with right- and goal-based issues would also still be exercised with regard to whether a person with few therapeutic options, such as those with severe PD, should be categorized as vulnerable in the same way that other patient populations are. Kipnis describes such a situation as ‘medical vulnerability’.^93^ No additional regulations exist for protecting such patients, although defining specific groups of patients as vulnerable is a task beset with conceptual and practical problems in any case, hence Kipnis' creation of a situation-based taxonomy of vulnerability.

Suggestions for addressing medical vulnerability through right-based ethical review include: requiring an impartial third party to approach potential participants and to conduct the informed consent process; requiring the provision of independent advocates with whom patients can discuss the research; avoiding approaching potential participants who have only recently learned that a standard treatment has failed; educating those enrolling patients not to overemphasize potential benefits nor underestimate risks; and finally, where possible, ensure that the investigator is not also the patient's own physician.^94^

Despite this, London and Kadane question whether researchers are best qualified to take responsibility for offering fair risk/benefit ratios in trials, given that they have an interest in putting scientific goals first.^95^ Emanuel, Wendler and Grady recommend independent review of the risks and benefits of research for similar reasons.^96^ As a rule of thumb, RECs might be charged with ensuring that the risks to which vulnerable persons are exposed would be acceptable to those who are not vulnerable in the same way.^97^

## EMPIRICAL STUDIES ON THE ETHICS OF SHAM SURGERY

With such strong duty-based, right-based and goal-based issues opposing one another, what other ethical perspective might be sought? Leeds calls for dialogue with the public, with people who have undergone sham surgery, with physicians and with surgeons.^98^ This dialogue is developing and in the next section we will turn to consider some of the main findings from empirical research on the subject of the ethics of sham surgery.

Kim et al.^99^ used quantitative and qualitative methods to explore the views of 103 members of the Parkinson Study Group, a group of Parkinson's disease experts in the US and Canada. In response to an online survey utilizing a hypothetical scenario, the vast majority of respondents believed that a sham-control design was scientifically superior to an open label design in an efficacy trial of a gene transfer therapy for Parkinson's. A similar majority thought that this was ethically permissible, while half questioned the ethics of an open label trial. The authors also found disagreement in the literature regarding the acceptable degree of invasiveness in sham surgery, noting that 22% of their respondents believed that a sham control with penetration of brain tissue was acceptable.^100^

These respondent scientists' apparent preference for goal-based arguments for the best (scientific) research methods is probably unsurprising. Indeed, the respondents might actually have been involved in the trials under consideration, although Kim et al. did not inquire as such. Another survey conducted by a research group, including scientists who have conducted a sham- controlled Parkinson's disease trial, also assesses North American researchers' attitudes, via focus groups and in-depth interviews.^101^ This group also concluded in support of sham-controlled trials and ‘challenged the custom of holding surgical trials to less stringent evidentiary standards’.^102^ Referring to Kim et al.'s findings, Olanow concludes that sham surgery is a ‘non-issue’ for researchers.^103^

Yet, as Kim et al. explicitly acknowledged, we also need to hear from other stakeholders. Accordingly, other members of their research team surveyed patients from a University-based neurology outpatient clinic and a community-based medical practice.^104^ Participants were given information about Parkinson's disease and two possible methods of testing the efficacy of a novel gene transfer protocol. Unblinded trials seemed preferable overall, but a majority (56% of 288 respondents) still supported the use of sham surgery. Patients with Parkinson's disease expressed less willingness to participate in the proposed surgery trials than those without. The authors thus conclude that the former group were more cautious about participation, although both groups held similar policy views on sham surgery. Frank et al. conclude that this caution may stem from patients with Parkinson's disease being better adjusted to their chronic illness than other patients in neurology or primary care, though an alternative view might be that the risks associated with sham surgery are more directly relevant, and more psychologically salient, to PD patients, hence their additional caution.

Notwithstanding certain limitations to their study (to which they are alert),^105^ in their discussion of their findings, Frank et al. state:

it appears that those who are supportive of sham controls are willing and able to understand and take on a societal perspective in giving their answers, whereas those who were opposed to it tend to focus on a more narrow view or simply state their disapproval.

Reviewing this conclusion in light of Foster's framework, however, it is possible that those opposed to sham surgery were more persuaded by duty-based concerns for the individual, while goal-based reasoning may have influenced those in favour of sham surgery. Frank et al conclude that:

this finding raises important questions about whether further intensive education and discussion about the scientific rationales and the actual risks and benefits of sham controls may affect people's opinions … Given the complexity of the topic, it may be that laypersons, especially those with less education, may need more opportunity to learn and deliberate on the issues.

Cohen et al.'s survey of patients' perspectives includes markedly contrasting opinions from two individuals with Parkinson's disease who have participated in clinical trials.^106^ One, a self-confessed ‘risk-taker’ who ‘likes to be on the cutting edge of research’ claims that she would enrol again today ‘because it gave me a few years of relief’; the other, an author on the paper, says ‘I'd have to think long and hard about going through the motions of having brain surgery and possibly not getting the treatment … that would seem emotionally and medically unethical’.

Interesting results also emerge from two surveys conducted online in 2007 and 2010, though it must be noted that these are ‘non-scientific’; being reported outside the peer reviewed academic publishing system and containing small and potentially non-representative samples. In 2007, 35 people with PD responded to a short online survey conducted by the Parkinson Pipeline Project. 36% of respondents thought that the scientific information gained by sham-controlled trials justified the risk to the volunteers who received sham surgery, while 38% stated they would be willing to participate in such a trial. A wide range of opinion was reflected in the data: amongst a wide range of open-ended comments was: ‘Scientific information gained? This sounds like something out of Nazi Germany’. Others were far more supportive of such experimentation: ‘It is impossible for me to adequately express my gratitude to those subjects who have participated in the trials involving control sham surgery. They are my heroes!’

When the Pipeline Project conducted a repeat survey in 2010, the differences in the results obtained compared with the previous survey were notable. Of 35 online respondents, 17% stated that they would volunteer for a trial that involved sham surgery, compared with 37% in 2007. Only 3% stated that they would volunteer for a trial in which brain tissue was penetrated in the sham group. When asked if the scientific information gained by clinical trials was worth the risk to trial participants who received sham surgery, 23% agreed, compared with 34% previously. 83% of respondents stated that they were aware of the use of sham controls before taking part in the survey, compared with 74% in 2007. The survey authors note the inconsistency between researcher opinions expressed in previous research and patient opinions in their survey.

## BACK TO THE FUTURE?

Neither the theoretical nor the small empirically-based literature reveals an easy route to broad consensus, and it is beyond the scope of this paper to ‘solve’ the ethical conflicts inherent in the use of sham surgery in neurodegenerative disease. Our intention has been to highlight that the ethical issues associated therewith have not gone away despite its continued use. The application here of Foster's framework for ethical review, however, adds clarity to the debate by showing us that sham surgery is supported by goal-based ethical arguments concerning methodology and benefits to science and future patients, by a duty-based argument of the presence of investigator equipoise, and by a right-based argument of respect for patient autonomy. Yet, at the same time, the method comes up against goal-based questions over the necessity of the methodology, duty-based concerns over participants' best interests when they are exposed to above-minimal harms and risks in the absence of a chance for benefit, and right-based arguments concerning the therapeutic misconception and the influence which degenerative chronic illness and restriction of treatments to trial may have on the actual autonomy of patients' decisions.

Added to these tensions are preliminary data reporting the attitudes of key stakeholders, which reveal that while researchers surveyed appear in favour of sham surgery, the views of patients with PD surveyed on the ethics of sham surgery appear more mixed.

In the context of a research method which carries above-minimal risks, it would seem wise for research ethics committees to ensure equal attention is paid to the goal-based, duty-based and right-based ethical issues surrounding sham surgery. The Parkinson's Pipeline Project calls for further investigation of patients' perspectives and more discussion of the issues. Our attempt to answer the former call is on-going;^107^ here, however, we hope to have contributed, in some measure, to the latter invitation.

## Footnotes

1 T.B. Freeman *et al.* Use of Placebo Surgery in Controlled Trials of a Cellular-Based Therapy for Parkinson's disease. *N Engl J Med* 1999; 341: 988–991.

2 Stereotactic surgery involves using images from CT or MRI scans to map the brain and provide location co-ordinates which are translated to a stereotactic frame fitted to the patient's head. This is used to guide an electrode (or cannula in the case of neurotransplantation) to the area of the brain to be treated. The stereotactic frame is affixed to the skull with metal pins and the electrode or cannula is inserted into the brain via a burr hole drilled through the skull. Such procedures may be performed under general or local anaesthesia. See R.A.E. Bakay, ed. *Movement Disorder Surgery*: *The Essentials*. New York, NY: Thieme Medical Publishers, Inc.

3 American Medical Association. *Code of Medical Ethics*. Available at: http://www.ama-assn.org/ama/pub/physician-resources/medical-ethics/code-medical-ethics.shtml[Accessed 16 Aug 2010]; American Association of Neurological Surgeons and Congress of Neurological Surgeons. 2000. *Position Statement on Placebo Surgery*. Available at: http://www.aans.org/Media/Article.aspx?ArticleId=9793[Accessed 15 Aug 2010].

4 M.K. Campbell *et al.* Effectiveness and Cost-effectiveness of Arthroscopic Lavage in the Treatment of Osteoarthritis of the Knee: a Mixed Methods Study of the Feasibility of Conducting a Surgical Placebo-controlled Trial (the KORAL study) *Health Technol Assess* 2010; 14(5).

5 For example World Medical Association General Assembly. 1964. *Declaration of Helsinki: Ethical Principles for Medical Research Involving Human Subjects*. http://www.wma.net/en/30publications/10policies/b3/17c.pdf[Accessed 2 Sep 2010]; National Bioethics Advisory Commission. 2001. *Ethical and Policy Issues in Research Involving Human Participants (Summary)*. Bethesda, MA; General Medical Council. 2002, *Good Practice in Research*; Medical Research Council. 2000. *Good Research Practice.* London: Medical Research Council; Royal College of Physicians. 2007. *Guidelines on the Practice of Ethics Committees in Medical Research*. Available at: http://bookshop.rcplondon.ac.uk/contents/pub232-e0da0967-8bed-4ac3-a12f-b05ad6a6c873.pdf[Accessed 2 Sept 2010];

6 Council of Europe. 1997. *Convention on Human Rights and Biomedicine*. Available at: http://conventions.coe.int/Treaty/Commun/QueVoulezVous.asp?NT=164&CL=ENG[Accessed 2 Sep 2010]; Council of Europe. 2005. *Additional Protocol to the Convention on Human Rights and Biomedicine, Concerning Biomedical Research*. Available at http://conventions.coe.int/treaty/en/treaties/html/195.htm[Accessed 26 Jul 2010].

7 C. Foster. 2001. *The Ethics of Medical Research on Humans*. Cambridge: Cambridge University Press.

8 Note there has also been a parallel debate regarding sham knee arthroscopy and other orthopaedic surgery. For the trials under question see J.B. Moseley *et al.* Arthroscopic Treatment of Osteoarthritis of the Knee. *Am J Sports Med* 1996; 24: 28–34; J.B. Moseley *et al.* A Controlled Trial of Arthroscopic Surgery for Osteoarthritis of the Knee. *N Engl J Med* 347; 2002: 31–88. For debate see, e.g. P. Angelos. Sham Surgery in Clinical Trials. *JAMA* 2007; 297: 1545–1546; D.R. Flum. Sham Surgery in Clinical Trials-Reply. *JAMA* 2007; 297: 1545–1546; S. Mehta *et al.* The Ethics of Sham Surgery in Clinical Orthopaedic Research. *J Bone Joint Surg* 2007; 89: 1650–1653. A.R. Vaccaro & M.G. Fehlings. The Applicability of Clinical Equipoise and Sham Surgery in Patients with Symptomatic Lumbar Radiculopathy due to a Herniated Disc. *Spine* 2007; 32: 2039–2040.

9 W.R. Brain & R. Bannister. 1992. *Brain and Bannister's Clinical Neurology*. Oxford: Oxford University Press.

10 R. Pahwa & K.E. Lyons. Levodopa-related Wearing-off in Parkinson's Disease: Identification and Management. *Current Medical Research and Opinion* 2009; 25: 841–849.

11 G. Fénelon & G. Alves. Epidemiology of Psychosis in Parkinson's Disease. *Journal of the Neurological Sciences* 2010; 289: 12–17.

12 For a full account of treatment options see the Parkinson's Disease section of the National Institute of Neurological Disorders website. Available at: http://www.ninds.nih.gov/disorders/parkinsons_disease/detail_parkinsons_disease.htm[Accessed 15 Aug 2010].

13 C.R. Freed. Transplantation of Embryonic Dopamine Neurons for Severe Parkinson's Disease. *N Engl J Med* 2001; 344: 71–719.

14 C.W. Olanow *et al.* A Double-blind Controlled Trial of Bilateral Fetal Nigral Transplantation in Parkinson's Disease. *Ann Neurol* 2003; 54: 403–414.

15 Ibid: 413

16 J.G. Nutt *et al.* Randomized, Double-Blind Trial of Glial Cell Line-Derived Neurotrophic Factor (GDNF) in *PD. Neurology* 2003; 60: 69–73. R.L. Watts *et al.* A Double-Blind Randomized Controlled Multicenter Clinical Trial of the Safety and Efficacy of Stereotaxic Intrastriatal Implantation of Fetal Porcine Ventral Mesencephalic Tissue (Neurocelltm-PD) vs Imitation Surgery in Patients with Parkinson Disease (PD). *Parkinsonism Relat Disord* 2001; 7: S87.

17 C.W. Olanow. Dopaminergic Transplantation for Parkinson's Disease: Current Status and Future Prospects. *Ann Neurol* 2009; 66: 591–596.

18 M. Politis. Serotonergic Neurons Mediate Dyskinesia Side Effects in Parkinson's Patients with Neural Transplants. *Sci Translat Med* 2010; 2: 38–46.

19 Available at: http://www.ceregene.com/press_112608.asp[Accessed 28 Apr 10].

20 A naturally occurring protein which facilitates the functioning of dopamine-secreting neurons.

21 Available at: http://www.ceregene.com/press-042810.asp[Accessed 28 Apr 2010].

22 For the associated Phase I trial see N.P. Stover & R.L. Watts. Spheramine for Treatment of Parkinson's Disease. *Neurother* 2008; 5: 252–259 which, as with other Phase 1 trials, had looked promising. No peer-reviewed Phase 2 data has been published at the time of writing.

23 P.D. Cohen et al. *‘Failed’ Clinical Trials Using Sham Brain Surgery Controls*. Available at: http://oba.od.nih.gov/oba/RAC/meetings/jun2010/SHAM%20surgery%20comments%20combined.pdf[Accessed on 05 Sep 2010]: 32–39.

24 See http://www.medicalnewstoday.com/articles/192625.php[Accessed on 15 Aug 2010]. For the associated Phase 1 study see M.G. Kaplitt *et al.* Safety and Tolerability of Gene Therapy with an Adeno-Associated Virus (AAV) Borne GAD Gene for Parkinson's Disease: an Open Label, Phase I Trial. *Lancet* 2007; 369: 2097–2105.

25 Food and Drug Administration. *Code of Federal Regulations* Title 21 Section 314.126. Available at: http://www.accessdata.fda.gov/scripts/cdrh/cfdocs/cfcfr/CFRsearch.cfm?CFRPart=314[Accessed 2 Sep 2010].

26 R. Tenery *et al.* Surgical ‘Placebo’ Controls. *Ann Surg* 2002; 235: 303–307.

27 Foster, *op. cit.* note 7.

28 See e.g. R. Adams. Internal-Mammary-Artery Ligation for Coronary Insufficiency. *N Engl J Med* 1958; 258: 113–116; T.L. Cobb *et al.* An Evaluation of Internal-Mammary-Artery Ligation by a Double-blind Technic. *N Engl J Med* 1959; 260: 1115–1118; J. Thomsen. Placebo Effect in Surgery for Meniere's Disease. *Arch Otolaryngol* 1981; 107: 271–277.

29 Freeman et al., *op. cit.* note 1.

30 G. Gillett. Unnecessary Holes in the Head. *IRB: Ethics and Human Research* 2001; 23: 1–6.

31 S. Polgar & J. Ng. Ethics, Methodology and the Use of Placebo Controls in Surgical Trials. *Brain Res Bull* 2005; 67: 290–297; A. Hrjóbartsson & P. Gøtzsche. Is the Placebo Powerless? – An Analysis of Clinical Trials Comparing Placebo with No Treatment. *N Engl J Med* 2001; 345: 1594–1602.

32 O. Rascol. Assessing the Risk of a Necessary Harm. *Neurology* 2005; 65: 982–983.

33 See e.g. G. Stirrat. Ethics and Evidence Based Surgery. *J Med Ethics* 2004; 30: 160–165; P. Mitchell. Equipoise and Randomisation in Surgery. *Br J Neurosurg* 2010; 24: 96–97; H. Buchwald. Surgical Procedures and Devices Should be Evaluated in the Same Way as Medical Therapy. *Control Clin Trials* 1997; 18: 478–487; R. McLeod *et al.* Randomized Controlled Trials in Surgery: Issues and Problems. *Surgery* 1996; 119: 483–486.

34 B. Freedman, K. Glass & C. Weijer. Placebo Orthodoxy in Clinical Research I: Empirical and Methodological Myths. *J Law Med Ethics* 1996; 24: 243–251; P. Lavori. Placebo Control Groups in Randomized Treatment Trials: A Statistician's Perspective. *Biol Psychiatry* 2004; 47: 717–723.

35 W. Dekkers & G. Boer. Sham Neurosurgery in Patients with Parkinson's Disease: Is it Morally Acceptable? *J Med Ethics* 2001; 27: 151–156.

36 A. Avins. Can Unequal be More Fair? Ethics, Subject Allocation, and Randomised Clinical Trials. *J Med Ethics* 1998; 24: 401–408; S. Edwards & D. Braunholtz. Can Unequal be More Fair? A Response to Andrew Avins. *J Med Ethics* 2000; 26: 179–182.

37 D. Machin. 2004. General Issues. In *Textbook of Clinical Trials*. D. Machin, S. Day & S. Green, eds. Chichester: John Wiley and Sons, Ltd: 11–44.

38 Dekkers & Boer, *op. cit.* note 35.

39 For Landau, the ethical risks for patients are best limited by longer, more thorough primate model studies. W. Landau. What is the Risk of Sham Surgery in Parkinson Disease Clinical Trials? A Review of Published Reports. *Neurology* 2005; 66: 1788.

40 Freeman et al., *op. cit.* note 1.

41 Ibid: 990.

42 N.M.P. King. Defining and Describing Benefit Appropriately in Clinical Trials. *J Law Med Ethics* 2000; 28: 332–343.

43 NBAC, *op. cit*. note 5.

44 Freeman, *op. cit*. note 1; Gillett, op. cit. note 30. Cf R. Rhodes. An Innovative Paradigm for Clinical Research. *Am J Bioeth* 2003; 3: 59–61; J. Fletcher. Sham Neurosurgery in Parkinson's Disease: Ethical at the Time. *Am J Bioeth* 2003; 3: 54–56.

45 R.E. Ashcroft. Equipoise, Knowledge and Ethics in Clinical Research and Practice. *Bioethics* 1999; 13: 314–326.

46 R. Macklin. The Ethical Problems with Sham Surgery in Clinical Research. *N Engl J Med* 1999; 341: 992–996.

47 We are aware of criticism of the use of a distinction between therapeutic and non-therapeutic research (see K. Lebacqz. Reflections on the Report and Recommendations of the National Commission: Research on the Fetus. *Vill L Rev* 1977; 22: 357–366; R.J. Levine. Clarifying the Concepts of Research Ethics. *Hastings Cent Rep* 1979; 9: 21–26); however, we follow Weijer's distinction between therapeutic procedures which are administered with the intent of providing direct benefit to subjects, and non-therapeutic procedures which are intended only to assist with answering the study question. See C. Weijer. The Ethical Analysis of Risk. *J Law Med Ethics* 2000; 28: 344–361.

48 C. Weijer & P.B. Miller. When Are Research Risks Reasonable in Relation to Anticipated Benefits? *Nature Med* 2004; 10: 570–573.

49 Council of Europe 2005, Article 17, *op. cit.* note 6; United States Department of Health and Human Services. *Code of Federal Regulations Title 45 Part 46.102*. Available at: http://www.hhs.gov/ohrp/humansubjects/guidance/45cfr46.htm#46.102[Accessed 3 Sep 2010].

50 Macklin, *op. cit.* note 46; P. Clark. Placebo Surgery for Parkinson's Disease: Do the Benefits Outweigh the Risks? *J Law Med Ethics* 2002; 30: 58–68.

51 Gillett, *op. cit.* note 30.

52 R. Albin. Sham Surgery Controls: Intracerebral Grafting of Fetal Tissue for Parkinson's Disease and Proposed Criteria for Use of Sham Surgery Controls. *J Med Ethics* 2002; 28: 322–325.

53 See e.g. S. Horng & F.G. Miller. Is Placebo Surgery Unethical? *N Engl J Med* 2002; 347: 137–139; F.G. Miller & H. Brody. Clinical Equipoise and the Incoherence of Research Ethics. *J Med Philos* 2007; 32: 151–165; F.G. Miller & D.L. Rosenstein. The Therapeutic Orientation to Clinical Trials. *N Engl J Med* 2003; 348: 1383–1386.

54 F.G. Miller. Sham Surgery: An Ethical Analysis. *Am J Bioeth* 2003; 3: 41–48: 42.

55 Ibid: 42.

56 C. Kowalski. Sham Surgery: Not an Oxymoron *Am J Bioeth* 2003; 3: W8–W9: W9; A.J. London. 2007. Clinical Equipoise: Foundational Requirement or Fundamental Error? In *Oxford Handbook of Bioethics*. B. Steinbock, ed. Oxford: Oxford University Press.

57 T. Lemmens & P.B. Miller. Avoiding a Jekyll-and-Hyde Approach to the Ethics of Clinical Research and Practice. *Am J Bioeth* 2002; 2: 14–17; D. Steinberg. Clinical Research Should Not Be Permitted to Escape the Ethical Orbit of Clinical Care. *Am J Bioeth* 2002; 2: 27–28.

58 P.B. Miller & C. Weijer. Equipoise and the Duty of Care in Clinical Research: A Philosophical Response to Our Critics. *J Med Philos* 2007; 32: 117–133.

59 See e.g. C. Clark. The Physician's Role, ‘Sham Surgery’, and Trust: a Conflict of Duties? *Am J Bioeth* 2003; 3: 57–58; C. Weijer. When Argument Fails. *Am J Bioeth* 2002; 2: 10–11; Steinberg *op. cit*. note 57; Lemmens & Miller *op. cit.* note 57; M. Kottow. Clinical and Research Ethics as Moral Strangers. *Arch Immunol Ther* Exp 2009; 57: 257–164; K. Glass & D. Waring. Effective Trial Design Need Not Conflict With Good Patient Care. *Am J Bioeth* 2002; 2: 25–26; D. Sprumont. 1998. Resolving the Inherent Dissonance Between the Doctor's Roles as Healer and Researcher: A Proposal. In *Research on Human Subjects: Ethics, Law and Social Policy*. D. Weisstub, ed. Oxford: Pergamon; Cf. D. Lenrow. The Treating Physician As Researcher: Is Assuming This Dual Role a Violation of the Nuremberg Code? *Temple J Sci Tech Env Law* 1996; 25: 15–48; Rhodes op. cit. note 44.

60 P. Angelos. Sham Surgery in Research: A Surgeon's View. *Am J Bioeth* 2003; 3: 65–66. This is the case even if the participant is in the placebo arm, since the placebo is intrinsically active rather than inert.

61 C. Clark *op. cit.* note 59.

62 S. Kim. The Sham Surgery Debate and the Moral Complexity of Risk-benefit Analysis. *Am J Bioeth* 2003; 3: 68–70.

63 Weijer *op. cit.* note 59.

64 I. Kennedy. 1988. Treat Me Right. Oxford: Clarendon Paperbacks. Foster *op. cit.* note 7; T.L. Beauchamp & J.F. Childress. 2009. *Principles of Biomedical Ethics*, sixth edn. Oxford: Oxford University Press.

65 I. Kant. 1785. *Groundwork of the Metaphysics of Morals*. In M. Gregor (translator). 1998. Cambridge: Cambridge University Press.

66 M. Parker, R. Huxtable & D. Dickenson. 2010. *Cambridge Medical Ethics Workbook*, second edn. Cambridge: Cambridge University Press.

67 Macklin, *op. cit.* note 46.

68 P.S. Appelbaum, C.H. Roth & C.W. Lidz. The Therapeutic Misconception: Informed Consent in Psychiatric Research. *Int J Law Psychiatry* 1982; 5: 319–329; C.W. Lidz, & P.S. Appelbaum. The Therapeutic Misconception: Problems and Solutions. *Med Care* 2002; 40(Supp): 55–63.

69 B. Ravina *et al.* Long Term Understanding of Study Information in Research Participants with Parkinson's Disease. *Parkinsonism Relat Disord* 2010; 16:60–63; M. Dixon-Woods *et al.* Beyond ‘Misunderstanding': Written Information and Decisions About Taking Part in a Genetic Epidemiology Study. *Soc Sci Med* 2007; 65: 2212–2222; M. Mangset. I Don't Like That, it's Tricking People Too Much …’: Acute Informed Consent to Participation in a Trial of Thrombolysis for Stroke. *J Med Ethics* 2008; 34: 751–756.

70 T. Grisso. 2001. *Method for a Study of the Therapeutic Misconception*. Worcester, MA: University of Massachusetts Medical School Center for Mental Health Services Research; D.T. Penman *et al.* Informed Consent for Investigational Chemotherapy: Patients' and Physicians' Perceptions. *J Clin Oncol* 1984; 2: 849–855; M. Schaeffer *et al.* The Impact of Disease Severity on the Informed Consent Process in Clinical Research. *Am J Med* 1996; 100: 261–268.

71 C.W. Lidz *et al.* Therapeutic Misconception and the Appreciation of Risks in Clinical Trials. *Soc Sci Med* 2004; 58: 1689–1697; S.L. Joffe. Quality of Informed Consent in Cancer Clinical Trials: A Cross-sectional Survey. *Lancet* 2001; 358: 1772–1777.

72 K. Featherstone & J.L. Donovan. ‘Why don't they just tell me straight, why allocate it?’ The Struggle to Make Sense of Participating in a Randomised Controlled Trial. *Soc Sci Med* 2002; 55: 709–719.

73 Gillett, *op. cit.* note 30.

74 Dixon Woods, *op. cit.* note 69.

75 N.E. Kass *et al.* Trust: The Fragile Foundation of Contemporary Biomedical Research. *Hastings Cent Rep* 1996; 26: 25–29:27.

76 Fletcher, *op. cit*., note 44.

77 While voluntariness in a legal sense is concerned with undue external influence upon a person's decision-making, we subscribe to Olsaretti's definition of voluntariness. See S. Olsaretti. Freedom, Force and Choice: Against the Rights-based Definition of Voluntariness. *J Polit Phil* 1998; 6: 53–78. Olsaretti argues that an individual's choice among options may be free so long as it is not subject to influence by other people. It may still be a non-voluntary choice, however, if it is made because no other option was acceptable other than the option ultimately chosen.

78 See e.g. D. Silverman. 1987. *Communication and Medical Practice: Social Relations and the Clinic.* London: Sage Publications; P. Atkinson. 1995. *Medical Talk and Medical Work*. London: Sage Publications.

79 R.R. Faden, T.L. Beauchamp & N.M.P. King. 1986. *A History and Theory of Informed Consent*. Oxford: Open University Press: 339; General Medical Council *op. cit.* note 5.

80 E.g. Dekkers & Boer, *op. cit.* note 35; Macklin, *op. cit.* note 46; Clark, *op. cit.* note 50.

81 For discussions of exploitation see D.B. Resnik. Exploitation and the Ethics of Clinical Trials. *Am J Bioeth* 2002; 2: 28–29. A. Wertheimer. 2001. Exploitation. Available at: http://plato.stanford.edu/entries/exploitation/[Accessed 24 Jul 2010].

82 Clark, *op. cit.* note 50.

83 Miller *op. cit.* note 54.

84 See S.J.L. Edwards *et al.* The Ethics of Placebo-controlled Trials: A Comparison of Inert and Active Placebo Controls. *World J Surg* 2005; 29: 610–614; S.J.L. Edwards. Restricted Treatments, Inducements and Research Participation. *Bioethics* 2006; 20: 77–91.

85 For example in trials of medication for psychiatric conditions. See e.g. J. Moncrieff, S. Wessely & R. Hardy. Meta-analysis of Trials Comparing Antidepressants with Active Placebos. *Br J Psychiatry* 1998; 172: 227–231.

86 Weijer *op. cit.* note 59.

87 P. Cohen *et al.* Ethical Issues in Clinical Neuroscience Research: A Patient's Perspective. *Neurother* 2007; 4: 537–544.

88 Ibid: 539.

89 Macklin, *op. cit.* note 46.

90 See S.J.L. Edwards, S. Kirchin & R. Huxtable. Research Ethics Committees and Paternalism. *J Med Ethics* 2004; 30: 88–91.

91 E. Garrard & A. Dawson. What is the Role of the Research Ethics Committee? Paternalism, Inducements, and Harm in Research Ethics. *J Med Ethics* 2005; 31: 419–423.

92 Foster, *op. cit.* note 7.

93 K. Kipnis. Seven Vulnerabilities in the Pediatric Research Subject. *Theor Med* 2003; 24: 107–120.

94 Online Ethics Center for Engineering. *Chapter 4: Assessing Risks and Potential Benefits and Evaluating Vulnerability (Research Involving Human Participants V1).* 14 June 2006. National Academy of Engineering. Available at: http://www.onlineethics.org/Topics/RespResearch/ResResources/nbacindex/nbachindex/hchapter4.aspx[Accessed 2 May 2011].

95 A.J. London & J.B. Kadane. Sham Surgery and Genuine Standards of Care: Can the Two Be Reconciled? *Am J Bioeth* 2003; 3: 61–64.

96 E. Emanuel, D. Wendler & C. Grady. What Makes Clinical Research Ethical? *JAMA* 2000; 283: 2701–2711.

97 Online Ethics Center for Engineering, *op. cit.* note 93.

98 H.S. Leeds. Social Aspects of Sham Surgeries. *Am J Bioeth* 2003; 3: 70–71.

99 S.Y.H. Kim *et al.* Science and Ethics of Sham Surgery: A Survey of Parkinson Disease Clinical Researchers. *Arch Neurol* 2005; 62: 1357–1360.

100 A majority would therefore presumably disagree with the sham group in the Neurologix trial which included cannula insertion into the brain and injection of saline solution.

101 A.W. Prehn *et al.* Studying Neurosurgical Implants for Parkinson Disease: A Question of Design. *Neurology* 2006; 67: 1503–1505.

102 Ibid: 1503.

103 K. Samson. Is Sham Surgery for Parkinson Disease Justifiable? *Neurology Today* 2005; 5: 5–7.

104 S.A. Frank *et al.* Ethics of Sham Surgery: Perspective of Patients. *Mov Disord* 2007; 23: 63–68.

105 E.g. the use of a hypothetical scenario; failure to assess illness severity of participants; response bias, through self-selection of participants, with the participants also not necessarily constituting a sample that is representative of the general public.

106 Cohen, *op. cit.* note 87.

107 The first author has conducted qualitative empirical research into UK patients' perspectives on participation in sham surgery in PD as part of a current PhD thesis: *The Ethical Implications of Sham Neurosurgery in Patients with Parkinson's Disease.*

